# The First Potentially Causal Genetic Variant Documented in a Polish Woman with Multiple Cavernous Malformations of the Brain

**DOI:** 10.3390/genes14081535

**Published:** 2023-07-27

**Authors:** Elżbieta Szczygieł-Pilut, Daniel Pilut, Michal Korostynski, Piotr Kopiński, Daniel P. Potaczek, Ewa Wypasek

**Affiliations:** 1 Department of Neurology with the Stroke Unit and Sub-Department of Neurological Rehabilitation, John Paul II Hospital, 31-202 Krakow, Poland; eszczygiel@vp.pl; 2 Department of Psychology and Psychopathology of Human Development, Faculty of Philosophy, John Paul II Pontifical University, 31-002 Krakow, Poland; 3 Individual Clinical Practice, 31-534 Krakow, Poland; pildan@poczta.onet.pl; 4 Laboratory of Pharmacogenomics, Department of Molecular Neuropharmacology, Maj Institute of Pharmacology, Polish Academy of Sciences, 31-343 Krakow, Poland; michkor@if-pan.krakow.pl; 5 Department of Lung Diseases, Cancer and Tuberculosis, Collegium Medicum, Nicolaus Copernicus University, 85-067 Bydgoszcz, Poland; mpkopins@hotmail.com; 6 Krakow Center for Medical Research and Technology, John Paul II Hospital, 31-202 Krakow, Poland; 7 Translational Inflammation Research Division & Core Facility for Single Cell Multiomics, Medical Faculty, Philipps University Marburg, 35043 Marburg, Germany; 8 Center for Infection and Genomics of the Lung (CIGL), Universities of Giessen and Marburg Lung Center (UGMLC), 35392 Giessen, Germany; 9 Bioscientia MVZ Labor Mittelhessen GmbH, 35394 Giessen, Germany; 10 Faculty of Medicine and Health Sciences, Andrzej Frycz Modrzewski Krakow University, 30-705 Kraków, Poland

**Keywords:** cerebral cavernous malformation (CCM), familial CCM (FCCM), programmed cell death 10 gene (*PDCD10* gene; *CCM3* gene)

## Abstract

Cerebral cavernous malformations (CCMs) are relatively common in the central nervous system. They occur in two forms, sporadic and familial (FCCMs). Three genes are recognized to be associated with FCCM, including *CCM1*, *CCM2*, and *CCM3*, the latter also called *PDCD10*. In this article, we describe a single-nucleotide variant in the *PDCD10* gene in a 23-year-old Polish female with CCM. The NM_007217.4 (*PDCD10*): c.395+1G>A variant destroys the canonical splice donor site following exon 6. This is the first reported genetically characterized case of CCM (FCCM) in Poland.

## 1. Introduction

Cerebral vascular malformations are a heterogeneous group of congenital pathologies within the vessels of the central nervous system. They vary in terms of structure and function, with approximately 15% of them being cavernous. Cerebral cavernous malformations (CCMs) are made up of dilated capillaries lacking sufficient structural support, such as elastic intima and smooth muscles. Inside them, a permanent process of blood clotting and clot lysis occurs. Weak vessel walls of CCMs favor bleeding [1]. The primary CCM symptoms comprise epileptic seizures, nonspecific headaches, dizziness, focal neurological deficits, and cerebral hemorrhage [2].

In the detection of CCMs, the imaging method of choice is magnetic resonance imaging (MRI) of the brain, including gradient echo (GRE) sequences or susceptibility weighted imaging (SWI). This imaging procedure can detect bleeding within the central nervous system, manifested by hemosiderin deposits [1]. When cavernous malformations are located in functionally important areas, diagnostics prior to planned neurosurgical intervention are often extended to include brain function tests. Examination using fiber tractography (FT) and/or diffusion tensor imaging (DTI) in the neuronavigation system during surgery, especially of lesions located within the brainstem, can be helpful in preventing the occurrence of severe postoperative neurological complications such as gait, balance, and sensory disorders [3]. Head computed tomography (CT) is not a useful diagnostic option. It often reveals an uncharacteristic radiological image that may inaccurately suggest a different nature of the pathology [4]. CCMs are classified as angiographically silent vascular dysfunctions [5]. Electroencephalography (EEG) is an auxiliary tool used when epileptic seizures occur. Genetic testing, especially in the case of multiple CCMs (see further), allows us to confirm the diagnosis [6].

Pharmacotherapy is in this pathology purely symptomatic, and medications are, for example, used to prevent the occurrence of epileptic seizures. Interventional treatment consists of surgical approaches, radiation therapy, or gamma knife, the latter dedicated to lesions located in the brainstem or other deep brain structures. Surgery is currently the only effective causal treatment, with seizures either decreasing or fully vanishing [7]. Standards of management for clinically silent cavernous malformations have not yet been established. However, there are a few widely accepted recommendations such as avoiding extreme sports or heavy physical exertion. Nevertheless, in the vast majority of cases, patients with the condition can lead a normal lifestyle [8].

CCMs occur in two forms, sporadic and familial (FCCMs). FCCMs comprise nearly 50% of all CCM cases in Hispanic Americans and close to 10–20% among white patients [9,10]. The diagnosis is established in a proband who has multiple CCMs or a single CCM and at least one family member with CCM and/or a heterozygous pathogenic variant [2]. FCCMs follow an autosomal dominant pattern of inheritance underlain by damaging variants in *CCM1*/*KRIT1* (chromosome 7q21.2) encoding a kyrin repeat containing protein, *CCM2*/*MGC4607* (chromosome 7p13) encoding CCM2 scaffold protein or *CCM3*/*PDCD10* (chromosome 3q26.1) coding for programmed cell death 10 (PDCD10), a protein associated with cell apoptosis [11]. However, recent reports suggest that in addition to *CCM1*-*3*, other genes might potentially carry variants underlying the disease, for instance *RNF213* [12]. Familial cases present a more aggressive phenotype, an increased risk of intracerebral hemorrhages and seizures, as well as an earlier age of onset [13].

To the best of our knowledge, the patient reported by us within this article is the first Polish case of FCCM.

## 2. A Case Report

Here we present the case of a 23-year-old female patient with mild mental retardation (62 points on the Wechsler IQ scale), who has remained under the care of an adult neurological clinic for periodic control of multiple cavernous malformations in the brain since she reached maturity.

The patient was subjected to brain MRI, performed with a 3T apparatus (Siemens Healthcare, Erlangen, Germany) in spin echo (SE) sequences T1, GRE 3D T1, SWI 3D T2, turbo SE T2, diffusion weighted imaging (DWI), and fluid-attenuated inversion recovery (FLAIR), before as well as after intravenous contrast administration. Sagittal, frontal, and transverse planes were recorded, with the smallest thickness of 1.5 mm.

**Figure 1:**
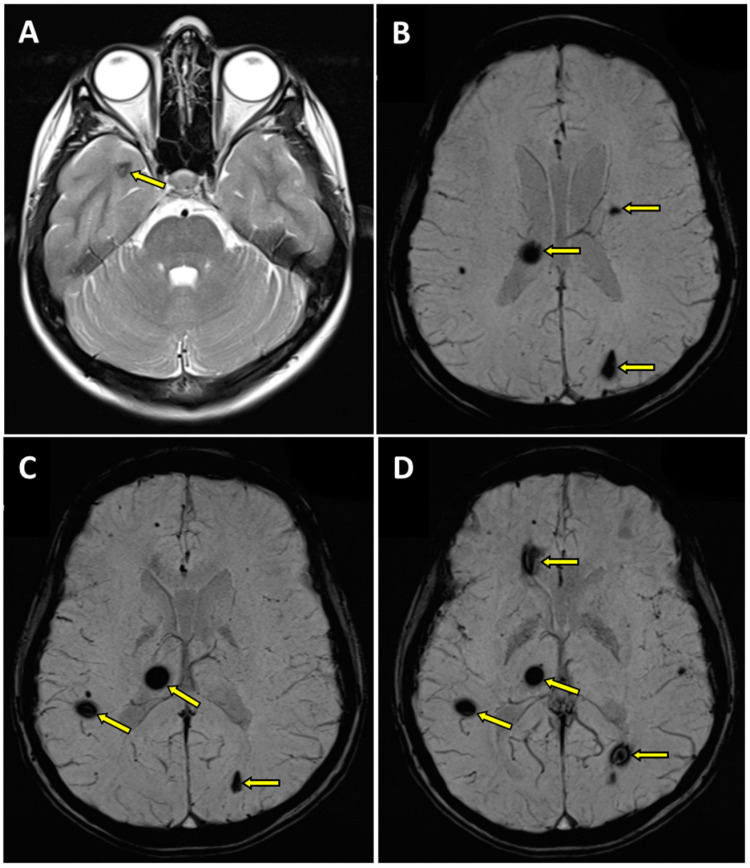
Magnetic resonance imaging (MRI) of the brain in T2 (**A**) and SWI (**B**–**D**) sequences. Transverse projection MRI showed the presence of cavernous hemangiomas within the right thalamus with hemosiderin deposits (**B**–**D**), at the fronto-parietal junction on the right side (**C**,**D**), in the semioval center on the left side (**B**), in the left occipital lobe (**B**–**D**), in the right temporal lobe (**A**), and in the right frontal lobe (**D**); pathologic changes marked with an arrow.

In the white matter and subcortex of frontal and parietal lobes, against the background of a few small (3–4 mm) foci of non-specific demyelination, the multiple vascular malformations of a cavernous type were found (Figure 1):

- within the right thalamus (transverse dimensions of approx. 6 × 3.5 mm), with hemosiderin deposits,
- at the fronto-parietal junction on the right side in the subcortical location (transverse dimensions of approx. 3 × 5 mm),
- in the semioval center on the left side (transverse dimensions of approx. 7 × 4 mm),
- within the left occipital lobe (transverse dimensions of approx. 10 × 6 mm),
- in the right temporal lobe (transverse dimensions of approx. 7 × 3 mm and approx. 8 × 5 mm),
- in the area of the right frontal lobe (transverse dimensions of approx. 10 × 6 mm).

In addition, minor diffuse changes of a similar nature were visualized in both cerebral hemispheres. Furthermore, discrete segmental enhancement without features of diffusion restriction was found at the border of the examined area, in the left-sided part of the medulla oblongata, after contrast administration (Figure 1). Those pathologies were first described in the patient at the age of 17.

**Figure 2:**
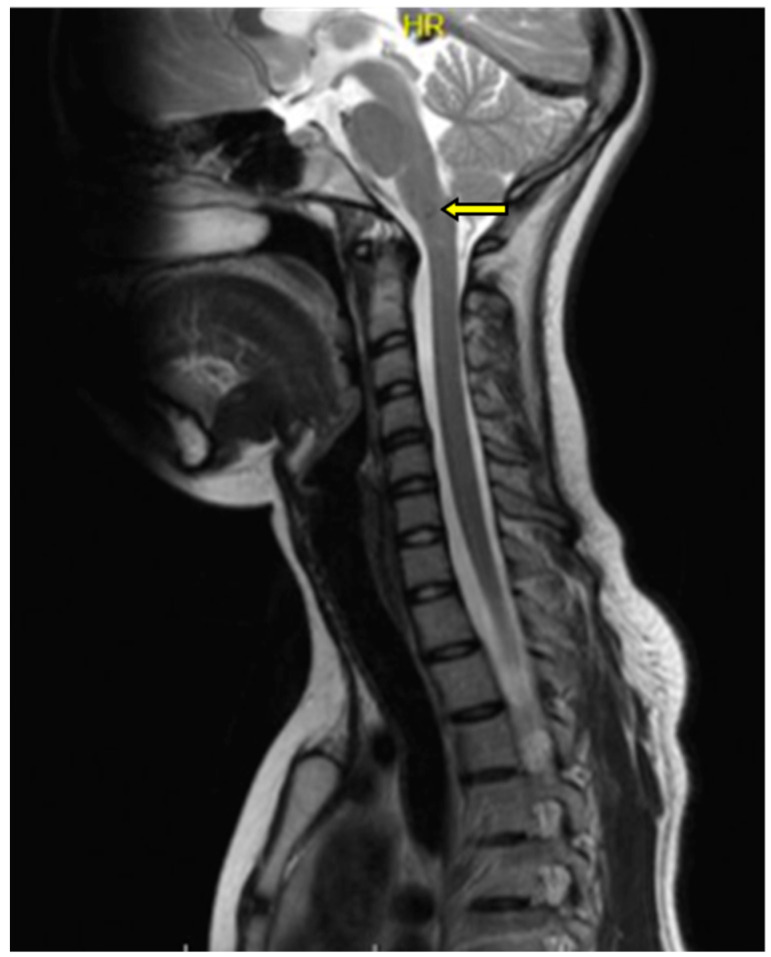
Magnetic resonance imaging (MRI) examination of the cervical spine in the T2 sequence. In the cervical spinal cord, no pathologies were seen. Within the medulla oblongata, a 2 mm lesion with a heterogeneous signal was visualized; pathologic change was marked with an arrow.

MRI of the cervical spine showed no changes in the spinal cord signal for the cervical or thoracic regions. In the medulla oblongata, at the border of the examined area, a 2 mm lesion with a heterogeneous signal in the T2 sequence was found (Figure 2).

Since the patient has remained in the care of the adult neurology outpatient clinic, the annual head MRI scan has shown no progression. It also has remained stable compared to childhood radiological procedures. The patient had no febrile convulsions in childhood.

In the neurological examination, apart from mild psychomotor retardation, no other abnormalities were found. There were no focal or meningeal signs.

**Figure 3:**
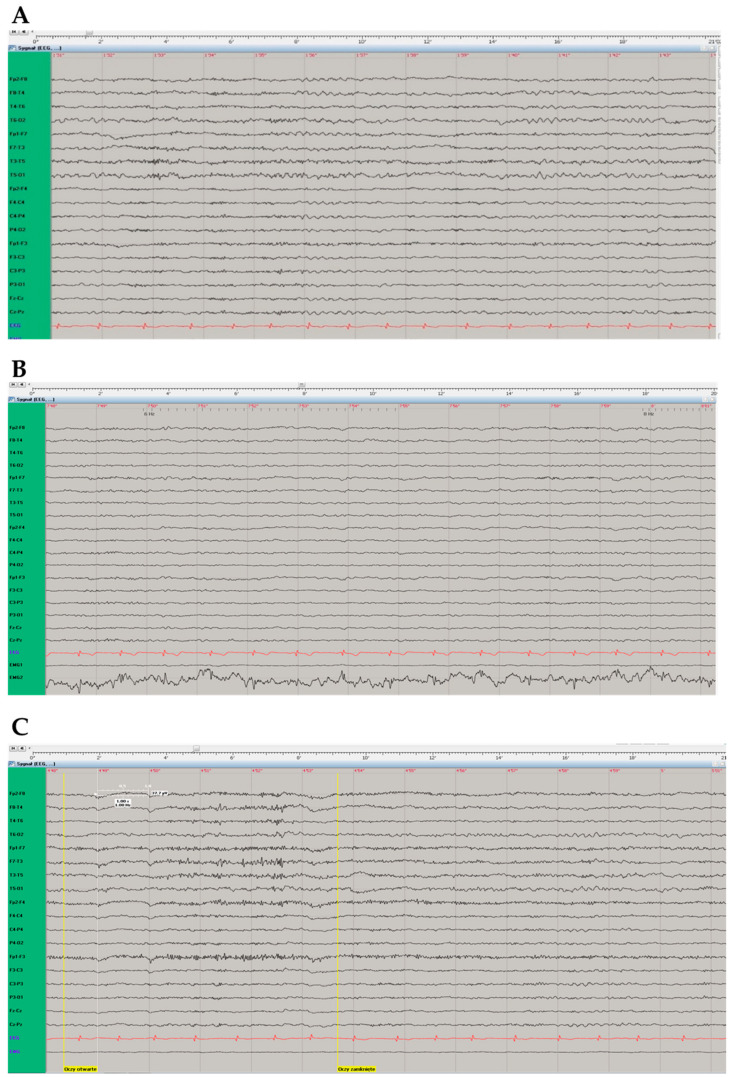
Electroencephalographic (EEG) examination. (**A**) “Double banana assembly”. EEG records without registration of paroxysmal changes, with registration of theta waves in the temporal and frontal-temporal leads, without clear lateralisation. (**B**) In photic stimulation during EEG record the background rhythm becomes time locked, representing a normal response. (**C**) Eyes-closed (“Oczy otwarte” [in Polish], left vertical yellow line) and eyes-open (“Oczy zamknięte” [in Polish], right vertical yellow line) reaction in EEG examination was normal.

In the video-EEG study paroxysmal changes were not observed. An increased share of theta waves (hyperventilation-activated, often generalising) against the background of marked disorganisation and an increased share of fast beta activity rhythms were primarily recorded in the temporal and frontal-temporal leads, without clear lateralisation (Figure 3).

Neuropsychological examination showed impaired auditory-verbal memory with preserved memory traces consolidation. Visual working memory was impaired. In terms of attention functions, weakening of visual-spatial functions including visual-motor coordination and letter fluency, as well as declined executive functions such as switching, cognitive flexibility, planning, and abstract thinking were observed. Activation functions have shown impaired alertness. The patient underwent neurosurgical consultation on several occasions, including at the reference gamma knife clinical center, and was disqualified from interventional treatment.

What is more, at school age, the patient was diagnosed with mild mitral regurgitation, thinned interatrial septum in its middle part, without signs of shunt at this level, and bronchial asthma. To date, the patient remains under regular care at the cardiology and pulmonology clinic. In addition, in indirect immunofluorescence testing (IIFT) on HEp-20-10 cells, moderately positive (a titer of 1:320) antinuclear antibodies (ANAs) with a granular/fine granular pattern were detected. However, further tests using an extractable nuclear antigen (ENA) panel failed to identify any specific antibody correlating with the observed IIFT ANA pattern. Furthermore, no cytoplasmic autoantibodies were detected in IIFT. Although moderately positive ANAs with a granular pattern represent a frequent unspecific and transient finding, the patient remains under the care of an immunologist. She does not receive any long-term medical or other types of treatment.

The patient was born from a pregnancy at risk for gestosis (the Apgar score was 5). On the mother’s side, one great-grandmother and grandmother had pacemakers implanted due to sick sinus syndrome (SSS). The family history on the father’s side is unknown, and the father came from the Mediterranean region.

## 3. Next-Generation Sequencing (NGS)

No approval of the bioethical commission was required, as this analysis was a part of a routine clinical and laboratory diagnostic evaluation process. After written informed consent for genetic testing was obtained, blood was drawn into K3-EDTA collection tubes and stored in aliquots at −80 °C until processing. DNA was extracted using the Sherlock AX DNA Purification Kit (A&A Biotechnology, Gdansk, Poland) according to the manufacturer’s protocol and stored at −80 °C until analysis. Whole exome sequencing (WES) was performed on the Illumina HiSeq 4000 (SanDiego, CA, USA) with an estimated mean coverage of 70×. Variant calling was filtered for those located in a custom gene panel formed by the user and composed of the 5112 genes related to cavernous angiomas.

## 4. Variant Annotation and Annotation-Based Filtering

Databases used for annotation included gnomAD v2.1 and v3 (frequencies, coverage, constraint), 1000Genomes (frequencies), MITOMAP (frequencies, contributed diseases), ClinVar (contributed diseases, pathogenicity), HPO (inheritance mode, contributed phenotypes and diseases), UCSC (repeats, PHAST conservation scores), SIFT4G (constraint), SnpEff (predicted impact on gene product), dbSNP (rsID), Ensembl (gene and transcript information), and COSMIC (somatic mutations data). Common and low impact variants were then filtered out (with maximal frequency threshold of 0.05 and minimal SnpEff predicted impact on gene product set as MODERATE). Annotated variants for genes that are likely to contribute to the patient phenotype and/or for genes from the user defined gene list/panels were then classified according to the American College of Medical Genetics and Genomic (ACMG) criteria [14] and prioritized.

## 5. Sanger Sequencing

The presence of a pathogenic variant was confirmed by Sanger sequencing. Briefly, primers for the polymerase chain reaction (PCR) and sequencing were designed using Pick Primers (https://www.ncbi.nlm.nih.gov/tools/primer-blast/index.cgi, accessed on 29 October 2021) software in a way making it possible to cover complete exon with at least 10 residues of their flanking intronic regions. The primer sequences for the *PDCD10* gene were as follows: forward 5′ ATGATTGAACGACCAGAGCC 3′ and reverse 5′ AGCATTTCAAGTTAGTCATACCAAA 3′. To amplify the chosen region, PCR with usage of HotStarTaq DNA polymerase (QIAGEN Benelux, Venlo, The Netherlands) and designed primers was performed. To confirm that the desired products were obtained, samples were subsequently separated by electrophoresis on 1.5% agarose gel. DNA bands were stained with SimplySafe dye (EURx, Gdansk, Poland) and compared to a GeneRuler 100 bp DNA ladder (Thermo Fisher Scientific, Waltham, MA, USA) size marker. Samples characterized with sufficient yield and specificity of the product were then purified using a prepared mix of Thermosensitive Alkaline Phosphatase and Exonuclease I (both Thermo Fisher Scientific). Purified PCR products were used as a matrix for direct Sanger sequencing performed using BigDye^®^ Terminator v3.1 Cycle Sequencing Kit (Thermo Fisher Scientific) and sequencing primers. After that samples were purified by ExTerminator (A&A Biotechnology, Gdansk, Poland) and dissolved in PCR-grade water. Following sequencing on the 3500 Series Genetic Analyzer, obtained chromatographs were analyzed using the SeqScape v2.6software (both Thermo Fisher Scientific).

## 6. Genetic Testing Results

**Figure 4:**
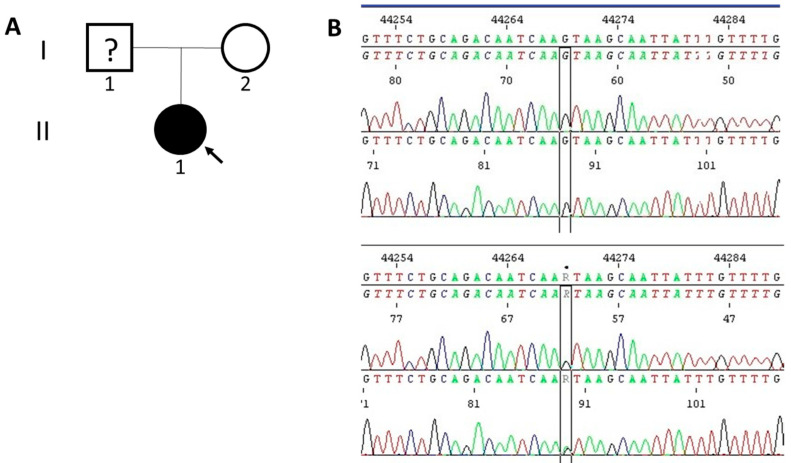
(**A**) The pedigree of the proband. The proband (II-1) is indicated with an arrow and marked with black as an affected individual. (**B**) The Sanger sequencing electropherograms of the proband (bottom) and her mother (upper).

Molecular analyses identified a potentially causal, heterozygous splice site variant NM_007217.4 (*PDCD10*): c.395+1G>A (rs1559952220) in our patient but not in her mother (Figure 4). This variant is interpreted as likely pathogenic according to the National Center for Biotechnology Information (NCBI) ClinVar (Genomic variation as it relates to human health) database (https://www.ncbi.nlm.nih.gov/clinvar/; accessed on the 12 June 2023) and pathogenic (11 points) in VarSome (The Human Genomics Community) variant knowledge community, data aggregator, and variant data discovery tool (https://varsome.com/; accessed on the 12 June 2023) germline variant classification, both referring to the ACMG Guidelines, 2015 [14]. Furthermore 12/12 VarSomeMeta Scores were calculated as pathogenic very strong or strong, while 13/15 VarSome Individual Predictions as pathogenic strong or supporting, and 2/15 as uncertain.

## 7. Discussion

Herein, we report the presence of the NM_007217.4 (*PDCD10*): c.395+1G>A (rs1559952220) variant in a patient with FCCM. To the best of our knowledge, this variant has never been described in the literature and no FCCM case report has originated from Poland so far. The prevalence of FCCM depends on ethnicity [9,10]. Our patient was of Polish descent in her maternal line, while her paternal ethnicity remains unknown. All we know is that her father came from one of the Mediterranean areas, where FCCM cases are relatively frequently reported [15]. Therefore, the described mutation should not necessarily be originally attributed to the Polish population and could derive from elsewhere, such as one of the Mediterranean countries. Interestingly, while the general proportions of FCCM families linked to *CCM1*/*KRIT1*, *CCM2*/*MGC4607*, and *CCM3*/*PDCD10* appear to be more evenly distributed, some large studies from Italy suggest *CCM2* and *CCM3* to be relatively underrepresented, at least in that population [16,17,18].

The NM_007217.4 (*PDCD10*): c.395+1G>A (rs1559952220) variant localizes within the canonical splice donor site following exon 6, specifically its first nucleotide (GT). Mutations destroying canonical splice sites prevent normal splicing, which can lead to the formation of transcripts including introns, partial deletions, or fully skipped exons, with or without the introduction of a premature termination codon (PTC), which yields truncated or otherwise non-functional protein [19,20,21]. Interestingly, the NM_007217.4 (*PDCD10*): c.395+2T>G (rs1559952217) variant is localized within the very same canonical donor site, in its second nucleotide (GT). Not surprisingly, the c.395+2T>G variant has been recently reported to affect *PDCD10* splicing [22]. Specifically, it has been shown that the c.395+2G allele leads to exon 6 skipping, ultimately resulting in the synthesis of a shorter protein losing the harmonic homology domain (HHD) as well as introduction of a PTC into the mRNA that is most likely targeted by a nonsense-mediated mRNA decay (NMD) process [22]. Indeed, NMD together with associated haploinsufficiency represent a typical mechanism underlying FCCM [23,24]. All those functional consequences are most probably associated also with the presence of the c.395+1A allele damaging the very same canonical splice donor site. The molecularly damaging and thus pathogenic character of the NM_007217.4 (*PDCD10*): c.395+1G>A (rs1559952220) gets further support from the in silico predictions reported in widely recognized clinical variation webpages such as VarSome and ClinVar using prediction software applying assessment algorithms based on ACMG criteria [14].

At present, we wish that other Polish patients with diagnosed cerebral cavernous hemangiomas would have the opportunity to deepen the molecular diagnostics of this pathology. This diagnosis should always be taken into account, especially in the case of multiple forms of these malformations. Additionally noteworthy is the fact that genetically confirmed mutations within the three genes characteristic for the CCM pathology have not yet been published in Poland. There is still no registry of families suffering from cerebral cavernous malformations in our country. In general, there are gaps in molecular testing for monogenic disorders, such as natural anticoagulant deficiencies [25] and Marfan syndrome [26], which need to be filled. In a more overall, international perspective, genetic testing is becoming one of the main inclusion criteria in clinical trials aimed at finding the optimal therapeutic approaches for FCCM [22,27,28,29]. Further studies, particularly clinical trials [27,28,29], along with more basic experimental investigations should clarify the molecular and cellular bases of CCM, identify new therapeutic targets [24,30], and thus help to optimize treatment strategies in affected subjects.

## Data Availability

The data presented in this work are available on request from the corresponding author.
